# Stereotactic body radiotherapy in the treatment of Pancreatic Adenocarcinoma in elderly patients

**DOI:** 10.1186/1748-717X-8-240

**Published:** 2013-10-16

**Authors:** Carolyn H Kim, Diane C Ling, Rodney E Wegner, John C Flickinger, Dwight E Heron, Herbert Zeh, Arthur J Moser, Steven A Burton

**Affiliations:** 1 Department of Radiation Oncology, University of Pittsburgh Cancer Institute, 5230 Centre Avenue, Pittsburgh, PA 15232, USA; 2 Department of Radiation Oncology, University of Pittsburgh Cancer Institute, 5150 Centre Avenue, #545, Pittsburgh, PA 15232, USA; 3 Department of Surgical Oncology, University of Pittsburgh Cancer Institute, # 413, Pittsburgh, PA 15232, USA; 4 Institute for Hepatobiliary and Pancreatic Surgery, Surgical Oncology, Beth Israel Deaconess Medical Center, 330 Brookline Ave, Boston, MA 02155, USA

## Abstract

**Background:**

Treatment of pancreatic adenocarcinoma in the elderly is often complicated by comorbidities that preclude surgery, chemotherapy and/or conventional external beam radiation therapy (EBRT). Stereotactic body radiotherapy (SBRT) has thus garnered interest in this setting.

**Methods:**

A retrospective review of 26 patients of age ≥ 80 with pancreatic adenocarcinoma treated with definitive SBRT+/-chemotherapy from 2007–2011 was performed. Twenty-seven percent of patients were stage I, 38% were stage II, 27% were stage III and 8% were stage IV. Patients most commonly received 24 Gy/1 fraction or 30-36 Gy/3 fractions. Kaplan-Meier was used to estimate overall survival (OS), local control (LC), cause specific survival (CSS) and freedom-from-metastatic disease (FFMD).

**Results:**

The median age was 86 (range 80–91), and median follow-up was 11.6 months (3.5-24.6). The median planning target volume was 21.48 cm^3^ (6.1-85.09). Median OS was 7.6 months with 6/12 month OS rates of 65.4%/34.6%, respectively. Median LC was 11.5 months, 6-month and 12-month actuarial LC rates were 60.1% and 41.2%, respectively. There were no independent predictors for LC, but there was a trend for improved LC with prescription dose greater than 20 Gy (p = 0.063). Median CSS was 6.3 months, and 6-month and 12-month actuarial CSS were 53.8% and 23.1%, respectively. Median FFMD was 8.4 months, and 6-month and 12-month actuarial rates were 62.0% and 41.4%, respectively. Nine patients (47%) had local failures, 11 (58%) had distant metastasis, and 7 (37%) had both. There were no acute or late grade 3+ toxicities.

**Conclusions:**

Definitive SBRT is feasible, safe and effective in elderly patients who have unresectable disease, have comorbidities precluding surgery or decline surgery.

## Background

Pancreatic cancer is the fourth leading cause of cancer-related deaths in the United States with an estimated incidence of 37,700 cases in 2008 [1]. Effective treatment remains a challenge, and prognosis remains poor. Most patients present with disseminated or locally-advanced disease, and less than 20% present with localized, potentially curable tumors [2]. Even after resections with curative intent, recurrence is common. The overall 5-year survival rate among pancreatic cancer patients is less than 5% [1].

Pancreatic cancer is more common in the elderly with 60% of patients presenting at or over the age of 65 [2]. Furthermore, the population of individuals over 85 years old experienced the fastest growth rate over the last decade, increasing by 29.9% [3]. In 2008, this disease was responsible for 10,779 deaths in patients age 80 and over [4].These trends warrant the importance of understanding management of diseases affecting the very elderly such as pancreatic cancer.

Surgical resection of the pancreas has been shown to be a safe option for some octogenarians, with rates of perioperative complications and mortality similar to those of younger populations [5]. For elderly patients with unresectable tumors, low-dose gemcitabine may improve prognosis [6]. However, elderly patients may have significant comorbidities that preclude surgery, chemotherapy, or a protracted course of external beam radiation therapy. Stereotactic body radiotherapy (SBRT) is a promising alternative modality as definitive treatment for these patients. SBRT has demonstrated encouraging results in the treatment of brain and spinal metastases, early stage bronchogenic cancer, prostate cancer, and head and neck cancer [7-10]. SBRT treatment for locally-advanced pancreatic cancer has also been shown to offer excellent local control with minimal toxicity [11,12]. We herein report the results of SBRT in the treatment of pancreatic adenocarcinoma in octogenarians at our institution, evaluating the safety and efficacy of this treatment modality.

## Methods

### Patient characteristics

Twenty-six patients age 80 years or greater with histologically-confirmed adenocarcinoma of the pancreas were treated in a definitive setting at our institution with SBRT between January 2007 and May 2011 (Table 1). Twenty-seven percent of the patients had stage I disease, 38% were stage II, 27% were stage III and 8% were stage IV. The two stage IV patients were treated with SBRT with palliative intent. Fourteen patients (54%) had locally-advanced disease extending into adjacent vascular structures rendering them unresectable, whereas 12 (46%) had resectable disease but either wished not to undergo surgical resection or were deemed poor surgical candidates due to their co-morbidities. Four patients (15%) received neoadjuvant chemotherapy, 6 patients (23%) received chemotherapy in the adjuvant setting, and 16 (62%) patients did not receive any chemotherapy. Immediately prior to receiving SBRT, 11 patients presented with abdominal pain, 5 with back pain, 5 with anorexia, 9 with weight loss, 11 with jaundice, and 2 with nausea.

**Table 1:** Patient and treatment characteristics

**Characteristics**	**No of patients (%)**
Median age (range)	86 (80–91)
Gender	
Female	19 (73)
Male	7 (27)
Stage	
I	7 (27)
II	10 (38)
III	7 (27)
IV	2 (8)
Chemotherapy	
Prior to SBRT	4 (15)
Post SBRT	6 (23)
None	16 (62)
Median PTV (range), cm^3^	21.48 (6.1-85.09)
Radiosurgery technique	
Truebeam IMRS	1 (4)
Cyberknife – SBRT	11 (42)
Trilogy – IMRS	14 (54)
Dose (Gy)/Fraction	
22/1	1 (4)
24/1	15 (58)
25/1	1 (4)
24/2	1 (4)
27/3	1 (4)
30/3	3 (11)
36/3	4 (15)

### Treatment planning and delivery

All patients were evaluated in a multi-disciplinary setting by surgical oncologists, radiation oncologists and medical oncologists to determine treatment recommendations based on tumor size, location, and extent of local disease. Patients were treated with Trilogy-Intensity Modulated Radiosurgery (IMRS) (Varian Medical Systems Inc., Palo Alto, CA), TrueBeam™-Intensity Modulated Radiosurgery (IMRS) (Varian Medical Systems, Palo Alto, CA) or Cyberknife robotic radiosurgery system (Accuray Inc., Sunnyvale, CA). Tumor movement as a result of respiration was managed with Synchrony respiratory tracking system (Accuray Inc., Sunnyvale, CA) with Cyberknife radiosurgery and Real-time Position Management Respiratory gating system (Varian Medical Systems Inc., Palo Alto, CA) with Trilogy IMRS and TrueBeam™ IMRS. All patients underwent 4D CT simulation to tract tumor motion, and gating was used on patients with >0.5 cm movement.

All patients treated with SBRT had endoscopically or percutaneously placed gold fiducials (2–4) placed in or near the tumor. Approximately 1 week after fiducial placement, a 4-dimensional CT (4D CT) of the abdomen with intravenous contrast was performed with the patient in the treatment position. For a precise and reproducible position, patients were immobilized with a customized Alpha Cradle (Smithers Medical Products, North Canton, OH) for Cyberknife or BodyFix™ patient positioning systems (Medical Intelligence Corporation, Pasadena, CA) for Trilogy-IMRS.

**Figure 1:**
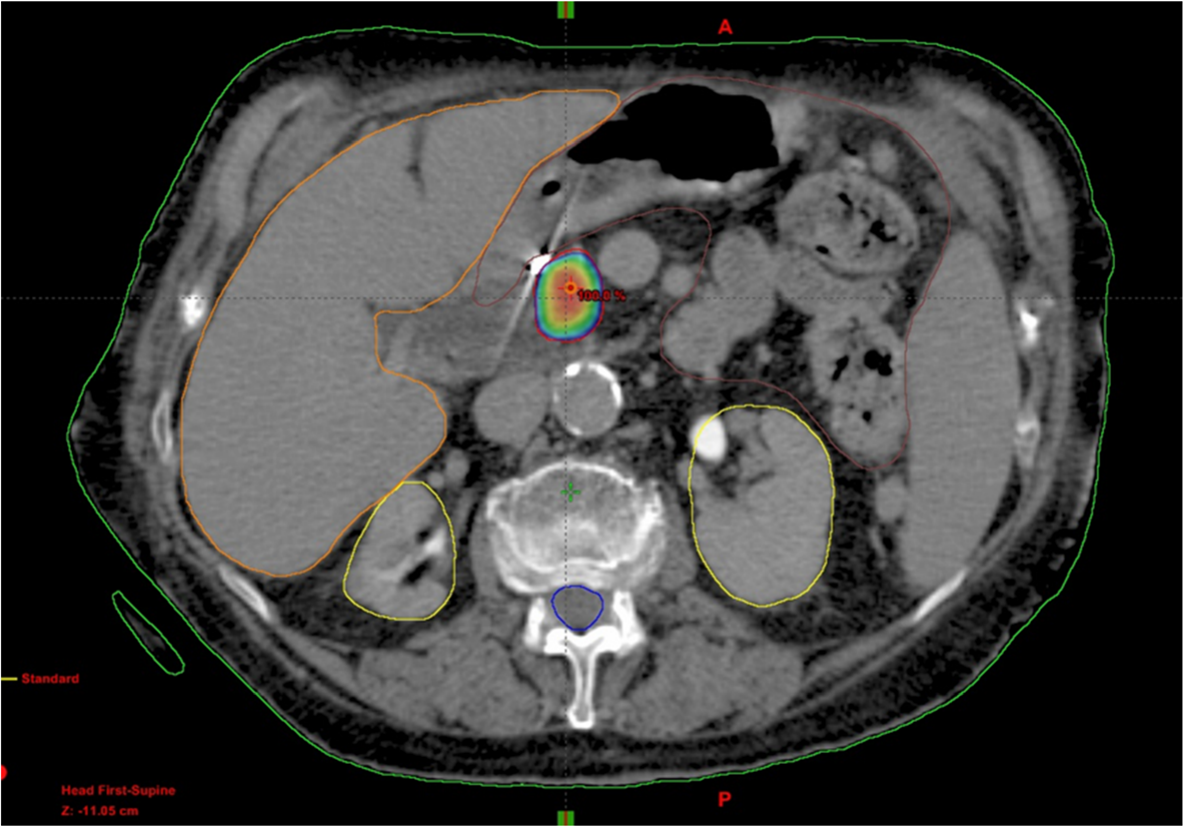
**Axial view of a stereotactic radiosurgery plan via TrueBeam™-IMRS (Varian Medical Systems, Palo Alto, CA) delivering 36 Gy given in 3 fractions to an 88 year old female.** PTV is shown in red. Blue colorwash outline shows 80% prescribed isodose line. Critical structures are outlined as follows: kidneys (yellow), liver (orange), bowels (brown), and spinal cord (blue).

The prescription dose and fractionation for each patient’s treatment regimen were determined by tumor size, location, goals of treatment, and patient’s performance status. A three-dimensional margin of 2 mm was added to the gross tumor volume to determine the planning target volume as shown in Figure 1. We prioritized limits of the maximum doses to critical structures such as small bowel, liver and stomach to ensure patient safety over optimal PTV coverage, assessed on a case-by-case as described in detail in a previous publication [12]. The median dose and range of doses to the small bowel, kidneys, liver, and spinal cord are listed in Table 2.

**Table 2:** Normal tissue dose constraints used for treatment planning

**Critical structure**	**Median maximum dose (Range)**
Liver	10.7 (4.1-24.5)
Right kidney	4.4 (1.7-12)
Left kidney	3.0 (0.7-12.9)
Small intestine	16.9 (10.4-29.9)
Spinal cord	3.8 (1.1-8.1)

Each patient was evaluated at the delivery of each SBRT fraction and 30 days after completion of treatment. Beyond 30 days, patients underwent regular follow-up by a member of the multidisciplinary team. Response to treatment was assessed by radiologists’ interpretation of contrast-enhanced CT scans or PET-CT obtained 2 to 3 months after completion and every 3 months until disease progression or death. For some patients, CA19-9 levels were also obtained at follow-up as well. When local progression was suspected but was questionable based on available imaging, clinical examination or laboratory values, biopsy was conducted. Improvement in any pre-treatment symptoms was based on clinical follow-up notes.

The primary endpoints of our study were local control (LC), overall survival (OS), symptom relief, acute and late toxicities, cause specific survival (CSS) and freedom from metastatic disease (FFMD). Local control was evaluated only in patients with at least one follow-up imaging, and local control was defined as stable or decrease in size byradiology report of CT or PET-CT scans with no new associated symptoms. Toxicities were scored according to the RTOG/EORTC grading criteria. Kaplan-Meier was used to estimate OS, LC, CSS and FFMD [13]. Univariate Cox regression analysis was conducted to identify significant predictors of the above outcomes [14]. Factors included in the Cox regression included target volume, age, sex, KPS, stage, fraction size, number of fractions, prescription dose, minimum dose, maximum dose, and whether or not the patient received chemotherapy.

### Treatment characteristics

The median SBRT dose was 24 Gy (24-36 Gy) prescribed to the 80% isodose line for Cyberknife, the 89% isodose line (80-93%) for Trilogy-IMRS, and 24 Gy prescribed to the 80% isodose line for TrueBeam™. Most patients (58%) received a single dose of 24 Gy, one patient received a single fraction of 22 Gy, one patient received 25 Gy, and the rest received 24-36 Gy in 2-3 fractions (Table 1). The most common fractionation schedule was 30-36 Gy given in 3 fractions, as this resulted in acceptable toxicities with the intended local control outcome in our experience. In general, the prescription doses were similar between definitively treated resectable and unresectable tumors.

### Chemotherapy

For patients receiving chemotherapy (n=13), gemcitabine was used in all cases. Four patients (15%) received chemotherapy in the neoadjuvant setting prior to undergoing SBRT, 6 (23%) patients received chemotherapy in the adjuvant setting, and 16 (62%) patients received no chemotherapy as they were unfit for systemic therapy based on medical co-morbidities.

## Results

**Figure 2:**
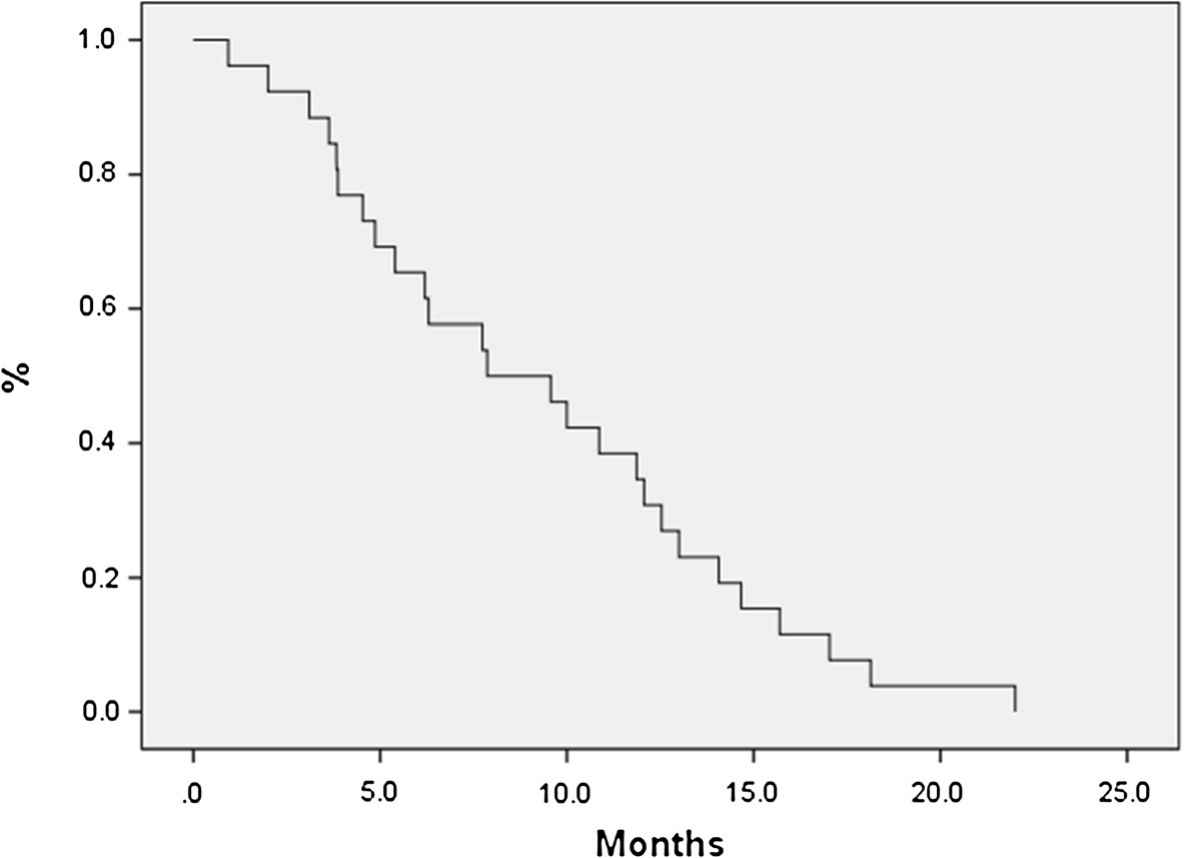
**Overall survival for all patients from time of SBRT.** The median OS was 7.6 months. Six-month and 12-month OS rates were 65.4% and 34.6%, respectively.

**Figure 3:**
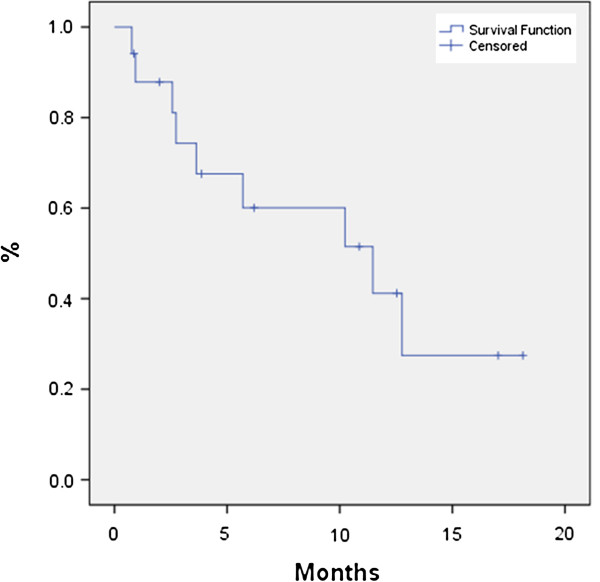
**Local control for all patients from time of SBRT.** Median LC was 11.5 months. Six-month and 12-month actuarial LC rates were 60.1% and 41.2%, respectively.

**Figure 4:**
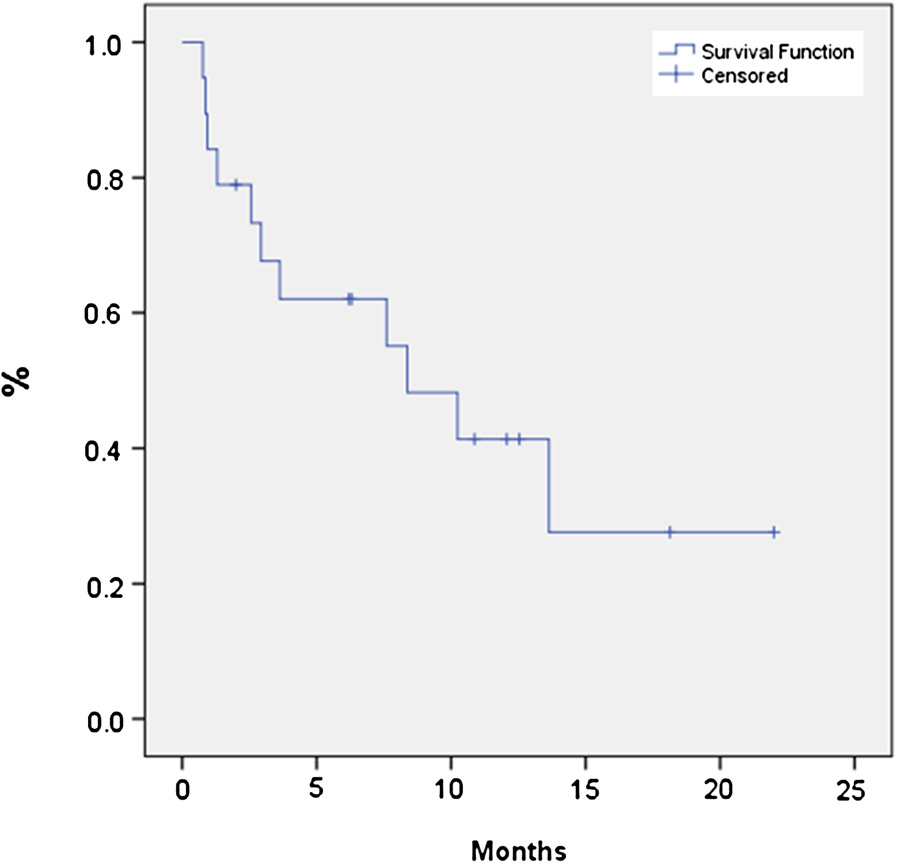
**Time to distant failure for all patients.** The overall median time of freedom from distant metastases was 8.4 months with a 6-month and 12-month actuarial rates of 62.0% and 41.4%, respectively.

**Figure 5:**
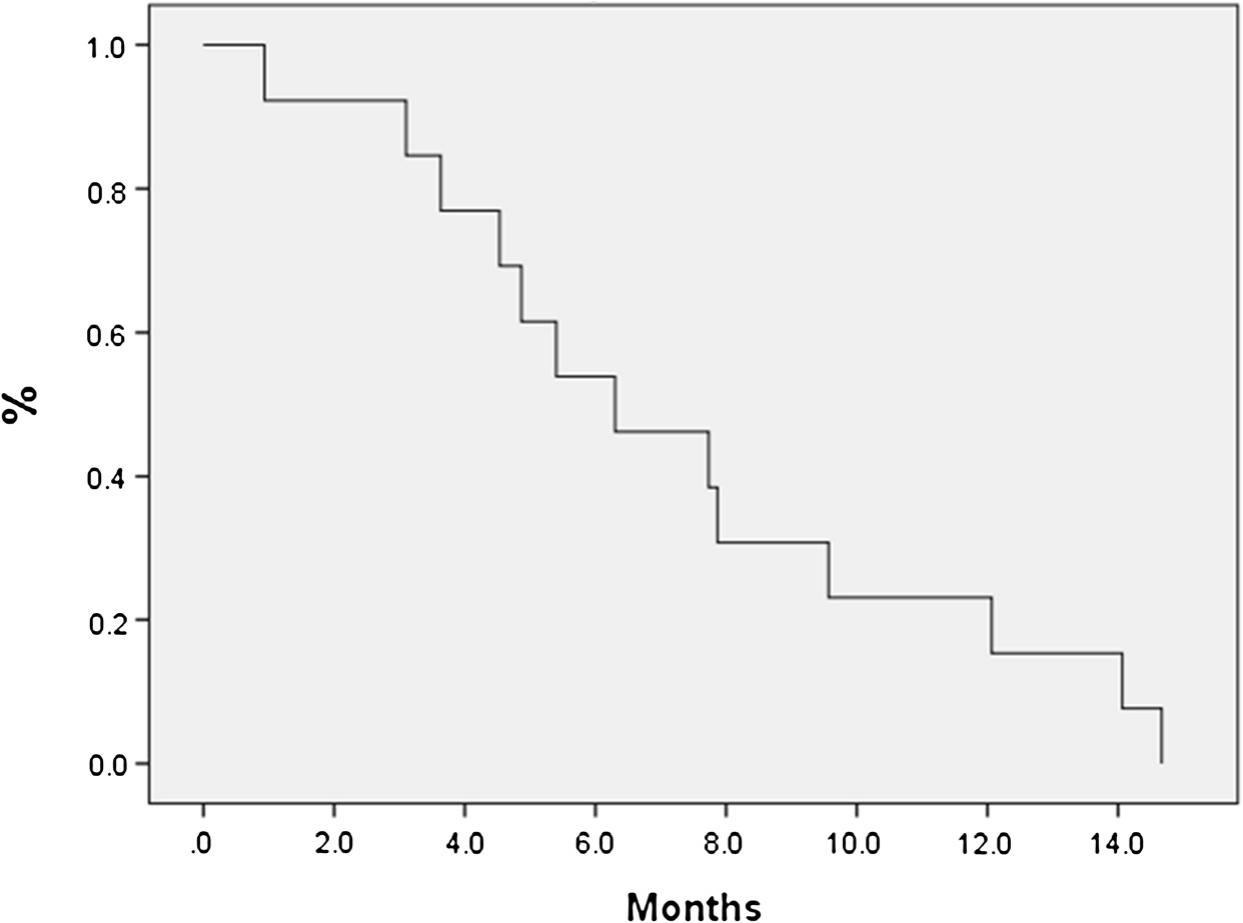
**Cause specific survival.** The overall median CSS was 6.3 months with a 6-month and 12-month actuarial rates of 53.8% and 23.1%, respectively.

The median age of patients was 86 (80–91) with a median follow-up of 11.6 months (3.5-24.6) from SBRT. Seventy-seven percent of patients (20/26) had follow-up imaging available for review. The median planning target volume was 21.48 cm^3^ (6.1-85.09 cm^3^). Most tumors exhibited minimal movement, and thus only 20% of the patients required gating. The median OS from time of SBRT was 7.6 months; 6-month OS was 65.4% and 12-month OS was 34.6% (Figure 2). Median LC was 11.5 months and 9 patients eventually developed local recurrence based on imaging, yielding a 6-month and 12-month actuarial local control of 60.1% and 41.2%, respectively (Figure 3). We did not identify any statistically significant independent predictors for LC; however, there was a trend for improved LC with prescription dose greater than 20 Gy (p = 0.063). Patients with resectable disease did not have significantly better local control (p=0.366) or overall survival (p=0.822) than those who were unresectable. Patients who received chemotherapy did not have improvement in LC (p=0.14), OS (0.71) or FFMD (0.15). The median time of freedom from distant metastases was 8.4 months with a 6-month and 12-month actuarial FFMD of 62.0% and 41.4%, respectively (Figure 4). Overall, 9 patients (47%) had local failures, 11 (58%) had distant metastasis, and 7 (37%) had both. The most common site of distant metastasis was the liver (50%). The median cause specific survival was 6.3 months with a 6-month and 12-month actuarial CSS of 53.8% and 23.1%, respectively (Figure 5). Only one patient was unable to complete treatment due to intractable pain, and there were no acute or late ≥ grade 3 toxicities.

Symptom relief was achieved in 8/10 patients who initially presented with abdominal pain (median time to response: 1 month), 3/4 patients with back pain (3 mo), 4/6 patients with anorexia (1.4 mo), 5/9 patients with weight loss (1 mo), 6/9 patients with jaundice (0.8 mo), and 2/2 patients with nausea (1.4 mo). Patients with weight loss were considered to have relief once patient stopped losing additional weight since completing treatment. Three patients with abdominal pain and 2 patients with back pain no longer required narcotic pain medication.

## Discussion

The incidence of pancreatic cancer increases with age with 60% of patients presenting over the age of 65. While surgery is the only treatment offering the possibility of cure, only 9-15% of patients are eligible for potentially curative resection [2]. Chemotherapy and/or radiotherapy are often the treatment of choice for patients with locally advanced or metastatic disease that is not amenable to surgical resection. Very elderly patients, however, may have significant comorbidities that preclude surgery, chemotherapy, or a protracted course of external beam radiation therapy. For these patients, stereotactic body radiotherapy (SBRT) is emerging as a promising new modality.

Multiple studies have found that age alone is not a contraindication to pancreatic resection [5,15,16]; however, both morbidity and mortality rates following pancreatic resection increase with advanced age. Elderly pancreatic cancer patients who undergo the Whipple resection have higher rates of postoperative complications such as delayed gastric emptying, pancreaticojejunal leak, sepsis, biliary leak, gastrointestinal tract bleeding, and intra-abdominal hemorrhage [17]. One study found that among patients who underwent pancreaticoduodenectomy, octogenarians had a mortality rate of 4.1% and a complication rate of 52.8% whereas these rates were 1.7% and 41.6%, respectively for younger patients [18]. Other conflicting studies have found that age did not significantly influence perioperative complications and mortality among pancreaticoduodenectomy patients [5,19]. However, the latter findings could be influenced by the stringent patient selection process applied to elderly patients chosen to undergo surgical resection.

Gemcitabine has been the first-line therapy for advanced pancreatic cancer for over the past decade [20], and Oettle et al. have shown that postoperative gemcitabine significantly delays disease recurrence after complete resection of pancreatic cancer compared with observation alone [21]. In a study of 68 patients, Matsumoto et al. found that low-dose gemcitabine may improve the prognosis of elderly patients (≥65) with unresectable pancreatic cancer compared to best supportive care (median survival 7.6 vs. 2.3 months) [6]. Disease progression occurred in 33% of patients and was stable in 53% of patients, while 15 patients had grade 3 or 4 toxicities. Another study of 30 patients aged ≥75 who underwent gemcitabine-based chemotherapy for advanced pancreatic cancer found a slightly higher median survival of 9.1 months. Disease control was obtained in 57% of patients, and grade 3 neutropenia was seen in 23% of patients with no grade 4 toxicities [22]. The literature on the efficacy of adjuvant chemoradiation therapy in elderly patients is somewhat limited. One retrospective series of elderly patients (defined as age ≥75) with pancreatic cancer treated with chemoradiation after pancreaticoduodenectomy, however, did show that elderly patients had improved 2-year survival with trimodality therapy compared with surgery alone (49.0% vs. 31.6%) [23].

We sought to evaluate the clinical feasibility of SBRT for the treatment of advanced pancreatic adenocarcinoma in octogenarians. Studies from Stanford University Hospital have suggested that SBRT for unresectable pancreatic adenocarcinoma is effective for local control but requires caution against the associated risk of toxicity [11,24,25]. Chang et al. reported that among 77 patients with unresectable pancreatic adenocarcinoma who received 25 Gy in 1 fraction, freedom from local progression (FFLP) rates at 6 months and 12 months were 91%/84% and overall survival rates at 6 and 12 months were 56%/21%. Median follow-up was 6 months and rates of grade ≥3 toxicity was 9% (7/17) [11]. Another Stanford study reported that among 16 patients with locally-advanced non-metastatic pancreatic adenocarcinoma, a single fraction of 25 Gy SBRT with gemcitabine resulted in good local control but significant late GI toxicities [26]. The overall survival at 1 year in this study was 50% with a median follow-up time of 9.1 months and 12.5% (2/16) grade ≥3 toxicity rate. Another series found SBRT to be feasible for the treatment of post-operative and advanced pancreatic adenocarcinoma with minimal grade ≥ 3 toxicity [12,27]. Our study confirms the feasibility, tolerability, and safety of SBRT for the treatment of primary or recurrent, locally-advanced or limited metastatic and unresectable cancers in the very elderly.

All local recurrences in this study were assessed by the radiologist’s report of the follow-up contrast-enhanced CT or PET-CT scans, but this presumes that the images are accurate in assessing local disease state. A recent study by Katz et al. studied the rate at which neoadjuvant chemotherapy or chemoradiation is associated with a reduction in the size or stage of borderline resectable tumors in 129 patients [28] with pancreatic cancer. CT scans obtained pre and post surgery were reviewed by faculty-level gastrointestinal radiologist to determine changes in tumor size or stage using modified Response Evaluation Criteria in Solid Tumors (RECIST) criteria. For 3/23 (13%) patients, there was a significant difference between the interpretation of CT images and intraoperative assessment. They were assessed as having stable disease per radiology; however, the operating surgeon found them to have locally advanced disease progression. In general, they concluded that there is no association between RECIST response and median OS duration. Similarly, we propose that CT or PET-CT images alone without a biopsy may also be less accurate in gauging local recurrence. For instance, one of our patients in a different study treated with definitive chemoradiation was assessed as having local recurrence per imaging report and subsequently underwent pancreaticoduodenectomy. This patient, however, was found to have a complete pathologic response. Therefore, our local control or local recurrence reported in our follow-up images may or may not reflect the true disease extent.

We previously reported 6-month and 1-year FFLP rates for metastatic, recurrent, and unresectable groups of 40%/40%, 56%/18.8%, and 63.4%/38%, respectively [12]. Our current study showed comparable rates with an overall 6-month LC of 60.1% and 1-year LC of 34.6%. We have also previously reported 6-month and 1-year FFLP rates of 94.7%/66% in a series of 24 patients with resected pancreatic carcinomas and close or positive margins [27] and overall FFLP rates at 6 months and 1 year of 100%/70.7% among 12 patients who had a Whipple with close margins (≤2 mm) [12]. One factor that may have contributed to our lower local control rate in this study is that we had a higher proportion of patients with locally-advanced adenocarcinoma. Therefore, it is not surprising that our overall LC rates were slightly worse than those reported in patients with resected disease, who had a better performance status, younger age, and perhaps less of a disease burden. Our results are also inferior to those reported by Chang et al. [11], who reported FFLP rate of 6-months and 12-months of 91% and 84%, respectively. This may be partially accounted for by the shorter median follow-up time of 6 months in that study compared to 11.6 months in our study. Other contributing factors may be the significantly higher proportion of patients (96%) in Chang et al.’s study who underwent chemotherapy, and the higher proportion of patients in our study who presented with regional lymph node involvement (at least 44%, compared with 19% in Chang et al.).

In Rwigema et al.’s study, which reported outcomes in 71 patients with locally-advanced pancreatic adenocarcinoma treated to a median dose of 24 Gy with 94% of the patients receiving a single fraction of 24 Gy, median FFMD was 2.8 months, 3.1 months, 3.0 months, and 9.7 months for metastatic, recurrent, unresectable, and post-Whipple, respectively [12]. The median time to distant metastases was similar in our study at 8.4 months.

Our median OS from time of SBRT was 7.9 months, which is comparable to the 10.3 months reported by Rwigema et al. [12]. Our 6-month and 12-month actuarial OS rates were 65.4%/34.6%, which is comparable to 57.4%/30.2% reported by Rwigema et al. [12] and 56%/21% in the Stanford study by Chang et al. [11], as well as previously published series of locally-advanced pancreatic adenocarcinoma [29,30].

SBRT was generally well-tolerated in this study. Only one patient was unable to complete treatment due to intractable pain, and there were no acute or late ≥ grade 3 toxicities. No patients had stenosis, ulceration, or perforation of bowel compared with 12% ulceration, 4% stenosis, and 1% perforation observed by Chang et al. [11]. Schellenberg et al. observed a significantly higher rate of late GI toxicities, including one grade 4 duodenal perforation [26]. Our study also observed lower toxicity rates than chemotherapy-only studies [5,23]. The lower toxicity rates seen in our study could be due to the use of fractionated treatments in 9 (35%) of our patients as opposed to the single fraction of 25 Gy that patients in Chang et al.’s study received, although no studies have compared single versus multiple fraction regimens. In addition, most of our patients did not receive chemotherapy, which could account for the decreased rate of toxicity. However, since all patients died within 2 years, the assessment of late toxicities in our study is likely limited as there may not have been enough time for these toxicities to manifest. We also showed that SBRT is effective in achieving symptom relief. Nine out of 11 patients who initially presented at the time of SBRT with abdominal pain and 3/5 patients who presented with back pain reported symptom relief after SBRT, which is comparable with the 13/16 patients in Rwigema et al.’s study who presented with symptoms of pain and reported complete pain relief shortly after SBRT [12]. In addition, we achieved symptom relief for most patients who presented with anorexia, weight loss, jaundice, or nausea. Further studies with larger sample size and longer follow-up may be warranted to perform more detailed assessment of pain control or symptom palliation, especially in patients treated with palliative intent.

Based on our experience, patient selection for definitive SBRT among octogenarians should take into account factors such as resectability, comorbidities that may complicate surgery, and potential symptomatic relief. However, one should note the small number of patients in this study when interpreting and applying our data. In select octogenarians who have either unresectable locoregional disease or resectable disease with comorbidities precluding surgery, we recommend definitive SBRT +/- gemcitabine.

## Conclusions

In an elderly population with co-morbidities and poor performance status that frequently preclude surgical management, SBRT is feasible, safe and effective. SBRT is a promising alternative to EBRT, offering a more convenient treatment regimen in a shorter overall treatment time while being better tolerated by patients. A prospective trial is currently underway to assess the efficacy of this approach.

### Consent

Written informed consent was obtained from patients for the publication of this report and any accompanying images.

## Competing interests

The authors declare that they have no competing interests.

## Authors’ contributions

The design of this study was HZ’s conception, and HZ made contributions in drafting and revising the manuscript. CK and DL acquired the data, drafted the manuscript and revised it critically. RW conducted the statistical analysis and made significant contributions in drafting and revising the manuscript. JF conducted the statistical analysis and interpretation of data. DW, HZ, AM, and SB made contributions in drafting and revising the manuscript. All authors read and approved the final manuscript.
